# Le goitre plongeant à Tlemcen dans l'ouest algérien: aspect clinique et thérapeutique de 1996-2014

**DOI:** 10.11604/pamj.2015.21.58.6615

**Published:** 2015-05-26

**Authors:** Smain Nabil Mesli, Derbali Regagba, Anisse Tidjane, Fouad Bouallou, Chakib Abi-Ayad

**Affiliations:** 1 Service de Chirurgie Générale A, CHU Tlemcen, Algérie; 2 Service de Chirurgie Hépatobiliaire et Greffe du Foie, EHU-1er Novembre 1954, Oran, Algérie

**Keywords:** Goitre plongeant, région cervicale, ouest algérien, Plunging goitre, cervical region, western Algeria

## Abstract

La définition du goitre plongeant, la plus couramment employée, est tout goitre ne siégeant pas dans la région cervicale en position opératoire. Le but d’évaluer sa prise en charge, en insistant plus particulièrement sur les examens préopératoires, les difficultés chirurgicales et les complications postopératoire. Etude rétrospective étalée sur 16 ans; portant sur 50 cas colligés au service de chirurgie viscérale du CHU Tlemcen. L'intervention a consisté en une thyroïdectomie totale par voie cervicale dans 94% des cas. Ont été notées essentiellement, les hématomes, des paralysies récurentiels et les hypoparathyroïdie. L’âge moyen est de 54,76 ±11,992 ans Le caractère plongeant du goitre à été retrouvé chez 52% (n = 26) par une échographie thyroïdienne. Le scanner cervico-thoracique était pratiqué chez 25 patients 50%. Il a permis de confirmer le caractère plongeant du goitre. Tous nos patients ont bénéficié eu un geste radicale (thyroïdectomie totale n = 45, 90%), alors que 5 patients (10%) ayant déjà ont eu un geste sur la thyroïde, ont subi une totalisation. L'examen histologique à confirmé la bénignité dans 48 cas 96%. Trois patients (6%) avaient une paralysie récurentielle transitoire et un cas (2%) de paralysie récurentielle persistante. L'hypoparathyroïdie transitoire était notée chez (14%). Les goitres plongeants représentent une éventualité assez fréquente. Souvent révélés à l'occasion d'un examen clinique. L'examen tomodensitométrique qui permet l'exploration des espaces peu accessibles à l'imagerie conventionnelle. L'imagerie par résonance magnétique nucléaire paraît constituer une technique d'avenir.

## Introduction

Le goitre plongeant ou substernal ou rétrosternal ou endothoracique a été décrit en premier par Haller en 1749 [1]. La définition du goitre plongeant reste à nos jours non unanimes [2]. La plus couramment employée, est considère comme plongeant tout goitre ne siégeant pas dans la région cervicale en position opératoire et ayant un prolongement inférieur à plus de deux travers de doigt sous le manubrium sternal [3]. Le goitre plongeant est souvent associé à une modification des hormones thyroïdiennes, surtout une élévation. Ce type de goitre est dit compressif, car il peut donner des signes respiratoires notamment une gêne due à la compression exercée sur la trachée. Ils posent un problème diagnostic [4] car, l'aspect clinique est rendu difficile par la morphologie du patient, avec des signes fonctionnels varies. Les examens préopératoires ont pour objectif d’évaluer le ou les prolongements endothoracique, pour prévenir au mieux les difficultés opératoires où la voie d'abord le plus souvent cervicale [5]. et parfois double cervico thoracique. A travers un travail réalisé au service de chirurgie viscérale « A » centre hospitalier universitaire Tlemcen « Ouest » (Chut), nous décrivons la prise en charge de ce type particulier de pathologie thyroïdienne, en insistant plus particulièrement sur les examens préopératoires, les difficultés chirurgicales et les complications postopératoire.

## Méthodes

Une étude rétrospective a été menée, portant sur tous les dossiers médicaux dans lesquels une thyroïdectomie pour goitre plongeant a été réalisée entre de juin 1996 à juin 2010 dans notre service. Il a été retenu comme critère de goitre plongeant, une extension inférieure de plus de deux travers de doigts sous le manubrium sternal patient en position opératoire [5]. Était inclus dans leurs dossiers médicaux pour l´étude. Les paramètres suivants qui ont été enregistrées et analysés: âge, le sexe, les antécédents pathologiques les signes fonctionnels le délai de consultation un examen général a été réalisé chez tous nos patients avec le statut hormonal pré et post opératoire. Concernant les examens paracliniques, il a été demande une radiographie thoracique, une échographie cervicale et un scanner cervico-thoracique afin de déterminer l'extension du goitre, et l'examen histologique définitif. Les dossiers médicaux, qui était incomplets ont étaient de l’étude. Sur le plan thérapeutique le traitement consistait une thyroïdectomie totale ou une totalisation pour les patients ayant subis une lobo-ismecthomie. La voie d'abord était une cervicotomie sous anesthésie générale, parfois il était nécessaire d'associer cette voies soit à une manibrutomie voir une thoracotomie. Les thyroïdectomies étaient toutes réalisées d´une manière similaire avec attention, dissection le long de la capsule de la thyroïde, tenté d´identifier et de préserver les glandes parathyroïdes ainsi que leur apport vasculaire, ainsi que les nerfs récurrents. La surveillance des malades était rigoureuse post opératoire à la recherche d’éventuels complication qui seront notées a savoir: suppuration de la plaie, les hématomes post opératoire, les paralysies récurentiels temporaires ou définitives et les hypoparathyroïdie transitoires ou définitives. Pour ce qui est de l'analyse statistique Les résultats ont été exprimés sous forme de fréquences et les pourcentages pour les données qualitatives et sous forme des moyennes ± écart type pour les données quantitative, et le Test Chi-deux comme moyen tous les calculs ont été effectués à l´aide de SPSS v13 (Chicago, IL) logiciel statistique.

## Résultats

Durant la période d’étude: 1850 cas de pathologie thyroïdienne ont étaient prise en charge au sein du service de chirurgie viscérale « A » Tlemcen et dont 50 cas de goitre plongeant soit un taux de 2,70%. Une nette prédominance féminine avec (84% Vs 16%) soit un sex-ratio de 0,2 l’âge moyen est de 54,76 ±11,992 ans avec des extrêmes «17-72ans. Le délai diagnostique était compris 02 mois à 56 mois avec un délai de prise en charge moyen 12,91 ± 9,1568 mois.

**Figure 1:**
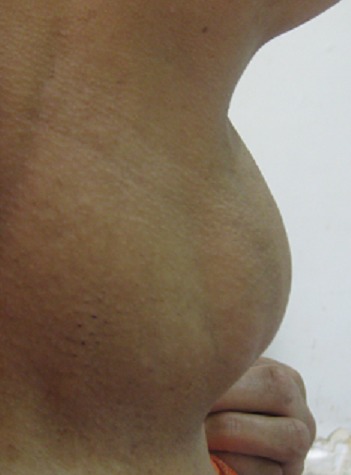
Vue de profil d'un goitre plongeant

**Figure 2:**
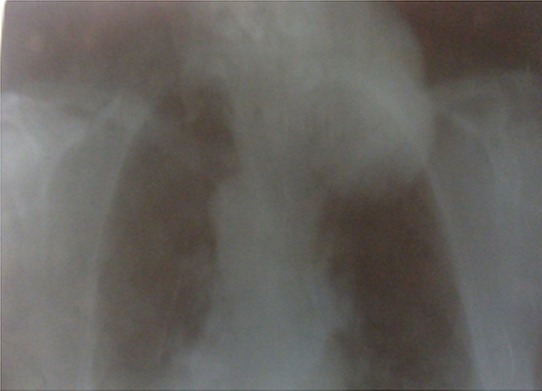
Radiographie du thorax: élargissement du médiastin antérieur avec déviation de la trachée vers la gauche

**Figure 3:**
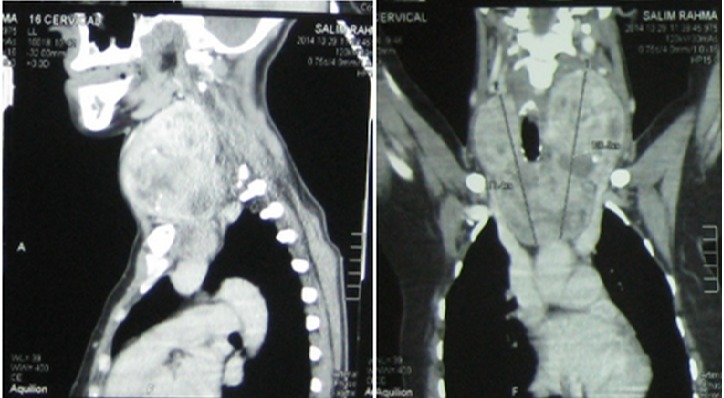
Aspect scanographique d'un goitre à développement antérieur vue de profil et de face

**Figure 4:**
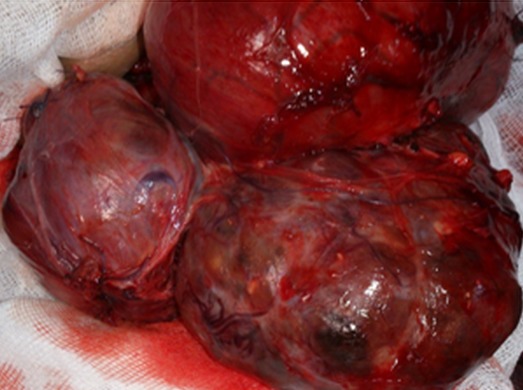
Vue per opératoire d'un goitre plongeant

La symptomatologie clinique était dominée par l'apparition des signes fonctionnels dans 76% des cas (n = 38) (Tableau 1). Ces signes étaient réparties en manifestations respiratoire dans 74% (n = 37) et qui se manifestaient à une simple gène respiratoire avec 46% (n = 23). Une toux dans 20% (n = 10) une dyspnée dans 16% (n= 8) et enfin 4% (n = 2) malades se traitaient pour maladie asthmatiforme pendant 10 ans. Par contre en dehors des signes respiratoires, nous avons des sensations de corps étranger avec augmentation du volume thyroïdien (Figure 1) dans 20% (n = 10), La dysphagie était présente dans 14% (n = 7). Enfin 12%, (n = 6) s'agissait d'une récidive d'un goitre nodulaire précédemment opéré sur un lobe. Par contre 24% des goitres plongeant étaient asymptomatiques et dont 10% (n = 5 cas) étaient de découvert per-opératoire. Pour ce qui est des bilans radiologiques, ceci a consistait à une radiographie du thorax dans 100% des cas (n = 50), mettant en évidence une opacité médiastinale dans 22% (n = 11) alors qu'une déviation de la trachée à était retrouvé dans 86% (n = 43)(Figure 2). L’échographie cervicale a montré un aspect hétérogène de la glande thyroïde pour 100% des cas (n = 50), alors que le caractère plongeant du goitre à été retrouvé que dans 52% (n = 26). Le scanner cervico-thoracique était pratiqué chez 50% (n = 25) de nos patients, il a permis de confirmer le caractère plongeant du goitre (Figure 3). Le bilan hormonal à été pratiqué chez tous nos malades celui-ci étant normal dans 94% (n = 47) le reste présentait une hyperthyroïdie 3 cas (6%), ce qui a nécessité un traitement hormonal en préopératoire. En ce qui concerne la prise en charge thérapeutique, la chirurgie à été réalisée dans 100% des cas, celle-ci consistait à une thyroïdectomie totale dans 90% des cas (n = 45) (Figure 4), alors que 5 patients (10%) ayant déjà ont eu un geste sur la thyroïde, ont subi une totalisation. La voie d'abord cervicale était suffisante dans 94% des cas (n = 47), celle-ci à été associée à une manubruotomie dans 4% (n = 2) des cas et enfin un cas 2% à nécessité une sternotomie programmée. Après extraction de la pièce opératoire celle-ci était adressée pour étude histologique qui a confirmé la bénignité dans 48 cas 96% alors que deux malades présentaient un carcinome papillaire thyroïdien. La taille moyenne du nodule plongeant était de 4,960 ±1,3845 cm avec des extrêmes de « 3-8cm ». Les suites post opératoire étaient marquées par la survenue de complications donnant un taux globale de morbidité de 18% (n= 9) (Tableau 2). 2% (n = 1) a présentait un hématome compressif qui a nécessité une reprise chirurgicale en urgence. Aucune complication respiratoire due à une trachéomalaçie n'a été noté ni aucune trachéotomie.

**Tableau 1:** Circonstance de découverte du goitre plongeant

Signes fonctionnels	Nombres	Pourcentage
Gene respiratoire	23	46%
Toux	10	20%
Dyspnée	08	16%
Maladie asthmatiforme	02	4%
Masse cervicale	10	20%
Dysphagie	02	14%
Récidive nodulaire	06	12%

**Tableau 2:** Répartition des complications post opératoires

Complications	Nombre de patients	Pourcentage
Hématome compressif	01	02%
Paralysie récurentiel transitoire	03	06%
Paralysie récurentiel persistante unilatérale	01	02%
hypoparathyroïdie transitoire	07	14%

Tous nos patients ont eu un control fibroscopique de la mobilité laryngée en post opératoire. Trois patients (6%) avaient une paralysie récurentielle transitoire et un cas (2%) de paralysie récurentielle persistante. Enfin aucune paralysie récurentielles définitives n'a été observée. L'hypoparathyroïdie transitoire a était notée chez 7 cas (14%), elle s'est manifestée par des fourmillements des membres et qui a persistée moins de 6 mois après supplémentassions en calcium. Aucune hypoparathyroïdie persistante n'a était observée. Aucun décès n'a était observé.

## Discussion

La définition d'un goitre plongeant n'est pas univoque [6] et plusieurs auteurs ont essayé de donner leurs propres définitions. la plus part d'entre eux définissent les goitres plongeants comme étant des goitres dont plus de 50% de leur masse totale se situe au dessous de l'orifice supérieur du thorax (Moran et Shen) [7, 8]. Makeieff pose trois critères pour définir le goitre endothoracique: siège au dessous de la région cervicale en position opératoire, un prolongement inférieur de plus de deux travers de doigt sous le manibrium, et la nécessité d'une manœuvre particulière lors de son extraction [5].

La définition la plus couramment employée et qu'on a retenu dans notre série est celle de Makeieff [5]. L'extension du goitre se fait préférentiellement vers la zone la moins résistante [9]. Le développement est soit antérieur ou postérieur par rapport au plan des troncs supra-aortiques, les goitres postérieurs sont les plus rares et représentent (10-15%) des goitres plongeants, [10] par contre les goitres antérieurs sont plus fréquent de par leurs situation rapidement compressif. La fréquence des goitres rétro-sternaux opérés est variable selon la littérature allant de 2-19% des thyroïdectomies [9–11] alors que Makeieff [5] rapporte dans sa série un taux de 7,8%. La série de M Rugui et al [12] un taux de 5,3% et que la série de Abboud et al [2] donne un taux de 12,35%. Ces données sont loin de notre série avec 2,7%. En faite, cela s'explique par la place de la pathologie thyroïdienne au quatrième rang dans sa prise en charge dans le service. La prédominance féminine est classiquement retrouvée dans la pathologie thyroïdienne. De ce fait le sexe féminin est prédominant dans les goitres plongeants dans notre série avec un taux de 84%. Ces données sont confirmées dans l'ensemble de la littérature [2–5]. L’âge moyen des patients rapporté dans certaines séries se situe au delà de la cinquième décade, ainsi la série tunisienne de R Zainine [13] rapporte un âge moyen (59,9ans), Abboud et al [2] (57ans) Rugui et al [12] (64ans) ce qui est le cas dans notre série avec (55,76. ans). Les signes communs les plus couramment rapportés dans la littérature sont les manifestations respiratoires (45%) [12] elle est retrouvée chez 73,94% de nos malades. Les manifestations respiratoires sont dominées par la dyspnée qui est présente dans (30% -48%) dans certaines séries [2–13], alors qu'elle est présente dans seulement dans seulement (16%) de nos malades, des taux aussi bas sont retrouvés dans les séries de Makeieff [5] et la série Pakistanaise de Mossadeque et Al [14] (12%- 17,6%).

Les autres manifestations respiratoire, tel la toux qui est rebelle aux traitements est présente dans 20% bien plus importante que la série de Makeieff [5] avec 5%. L'augmentation de la douleur de la région cervicale, ainsi que la sensation de corps étranger que Makeieff [5] rapporte dans sa série est de l'ordre 42%. La série indienne donne un taux de 75% [15], alors que nous nous avons que seulement 20% dans la notre. Par ailleurs la dysphonie, de fréquence variable selon la littérature ou R Zainine et al [13] rapporte un taux de 11, 6%, 37% pour J Rodrigues et al [15] Makeieff et al [5] 14,1%, par contre on ne note aucune dysphonie dans notre série. D'autre part, la dysphagie due le plus souvent à une compression de l’œsophage révélant un goitre plongeant chez 14% de nos malades alors que dans d'autres séries ce taux varie de 31,6% à 11% [2–15].

Enfin comme toute pathologie thyroïdienne, l'hyperthyroïdie peut être la première manifestation clinique sa fréquence avoisine les 15% [2], elle était révélatrice dans 6% des cas dans notre série, ce qui à nécessité chez ces malades un traitement à base d'antithyroïdien de synthèse et des betas bloquant avant toute acte opératoire. L'examen clinique, est un temps capital qui permet d'apprécier la taille du goitre et va suspecter son caractère plongeant, cependant il n'est pas toujours facile en fonction de la morphologie du patient (obésité, cou court). Pour ces raisons, le recourt aux examens morphologique est indispensable. La radiographie standard du thorax est l'examen le plus rentable qui garde un rôle irremplaçable dans le dépistage des goitres plongeants [16]. Il permet d'objectiver une opacité médiastinale et/ou une déviation de la trachée. Cette dernière est le signe le plus fréquemment observé et varie de 53.5% à 84.9% [13, 14], il est présent chez 87.15% de nos malades. L’échographie cervicale confirme la nature thyroïdienne de la tuméfaction cervicale, mais elle ne permet pas d’évaluer le prolongement thoracique. Dans notre série, cet examen a été réalisé chez tous les malades (100%) et a permis de détecter le caractère plongeant du goitre chez 26 malades (52%). En cas de doute du prolongement thoracique du goitre, c'est le scanner cervico thoracique qui va l'affirmer, apprécier son volume, son contenu liquidien ou solide, sa position par rapport à la trachée, vaisseaux et l’œsophage [4–13]. La plupart des auteurs rapportent une sensibilité optimale pour ces examens [17] ce qui est rencontré effectivement dans notre série puisque 50% de nos patients ont bénéficié de cet examen. On ne note pas de faux négatif et par conclusion la TDM est considérée actuellement dans les nouvelles publications comme le Gold-Standard dans les investigations radiologiques en pré opératoire [18]. Quant à l'imagerie par résonance magnétique, son intérêt est d'obtenir des coupes frontales et sagittales. Dans le plan frontal, on visualise les rapports avec le tronc brachio céphalique, de l'artère sous Clavière et carotide interne. Il est en revanche plus difficile de définir les rapports veineux [4].

La prise en charge thérapeutique des goitres plongeants est exclusivement chirurgicale car plusieurs études ont prouvé l'inefficacité du traitement médical (hormonothérapie ou Irathérapié) [19]. L'indication opératoire est posée devant le risque de compression, d'hyperthyroïdie et devant le risque de dégénérescence. Le but est de réaliser une exérèse radicale du goitre la moins invasif possible avec une morbidité réduite. Pour cela l'abord cervical suffit dans la majorité des cas [2] 94% de nos malades ont eu un abord cervical seul, la sternotomie et la manubriotomie associées ne sont indiquées qu'après échec de l'extraction du nodule intra thoracique par voie cervicale. Sa fréquence est variable entre 2% et 12% [20]. Dans notre série, nous avons 4% de manubriotomie (2 cas) et 2% de sternotomie (1 cas).

L'importance de la thyroïdectomie est dictée par la pathologie. Le plus souvent une thyroïdectomie totale ou une totalisation est réalisée chez nos malades. L'intervention s'est déroulée sans grande difficulté technique, le prolongement thyroïdien médiastinal a été extrait facilement au doigt après ligature du pole supérieur et repérage du nerf récurrent et des parathyroïdes inférieures. La morbidité globale est variable et avoisine les 4% [7], le taux de morbidité dans notre série est de 18%. L'hématome compressif est la principale urgence post opératoire immédiate, il peut être responsable de détresse respiratoire brutale. C'est le cas dans 2% des cas de notre série alors que dans la littérature, il est de 1.5% pour Makeieff [4]. En revanche aucun de nos patients traités n'a présenté des troubles respiratoires post opératoire en rapport avec une trachéomalacie qui peut entrainer une réintervention voir une trachéotomie après cicatrisation de la cavité. Le risque récurrentiel dans la chirurgie des goitres plongeants apparait supérieur à celui de la chirurgie thyroïdienne conventionnelle, sa fréquence est variable selon les auteurs, 2% [14]. Pour notre part, le taux de paralysie récurrentielle unilatéral transitoire est de 16%, par contre la paralysie unilatérale définitive est de 2% (1 cas).

Les glandes parathyroïdes sont ainsi exposées à un traumatisme chirurgical, elles sont facilement lésées lors de l'extraction des goitres. L'hyporparathyroïdie postopératoire immédiate est très fréquente en cas de geste bilatéral est s'améliore généralement rapidement sous traitement médical. Son incidence avoisine les 22.5% [13]. Dans notre série l'hypoparathyroïdie postopératoire est retrouvé chez 4% de nos patients et aucun cas d'hypoparathyroïdie définitive. En ce qui concerne la mortalité post opératoire, l'ensemble des séries rapportent des taux variables entre 0 et 1.3% [2], elle est nulle dans notre série. L'incidence des carcinomes développés sur un goitre plongeant peut aller de 1.3 à 3.7% [20]. Dans notre série, nous avons un taux de 2% de cancer papillaire.

## Conclusion

Les goitres plongeants représentent une éventualité assez fréquente. Ils restent pendant plusieurs années asymptomatiques et sont le plus souvent révélés à l'occasion d'un examen clinique ou radiologique systématique, leur mode d'expression peut être plus bruyant, inhérent à une compression des structures de voisinage. L'imagerie thoracique et cervicale standard peut révéler une masse médiastinale supérieure. C'est surtout l'examen tomodensitométrique qui permet l'exploration des espaces peu accessibles à l'imagerie conventionnelle et l'analyse des structures de voisinage, particulièrement de la trachée. L'imagerie par résonance magnétique nucléaire paraît constituer une technique d'avenir. Le diagnostic de goitre thoracique impose une exérèse chirurgicale, le plus souvent par voie cervicale pure, plus rarement par sternotomie. Les arguments en faveur de ce traitement chirurgical sont doubles: d'une part l’évolution inéluctable vers des phénomènes compressifs; d'autre part, l'hormonothérapie frénatrice ayant peu d'impact sur l’évolution des goitres volumineux. Une technique chirurgicale réglée et codifiée constitue le meilleur moyen pour éviter les complications nerveuse et endocrinienne.

## References

[1] Haller A (1749). Disputatones Anatomica Selectae.

[2] Abboud B, Sleilaty G, Mallak N (2010). Morbidity and mortality of thyroidectomy for substernal goiter. Head Neck..

[3] Barrault S, Gandon J, Le Guillou C (1986). Les goitres plongeants et médiastinaux. Annales d'oto-laryngologie et de chirurgie cervico-faciale.

[4] Makeieff M, Marlier F, Khudjadze M (2000). Substernal goiter; report of 212 cases. Ann Chir..

[5] De Perrot M, Fadel E, Mercier O (2007). Surgical management of mediastinal goiters: when is a sternotomy required?. Thorac Cardiovasc Surg..

[6] Agha A, Glockzin G, Ghali N (2008). Surgical treatment of substernal goiter: an analysis of 59 patients. Surg Today..

[7] Moron JC, Singer JA, Sardi A (1998). « Retrosternal goiter: a six-year institutional review ». The American Surgeon.

[8] Shen WT, Kebebew E, Duh Q-Y (2004). Predictors of airway complications after thyroidectomy for substernal goiter. Arch Surg.

[9] DeSouza FM, Smith PE (1983). Retrosternal goiter. J Otolaryngol..

[10] Mack E (1995). Management of patients with substernal goiters. Surg Clin North Am..

[11] Wu M-H, Chen K-Y, Liaw K-Y (2006). Primary intrathoracic goiter. J Formos Med Assoc..

[12] Rugiu MG, Piemonte M (2009). Surgical approach to retrosternal goitre: do we still need sternotomy?. Acta Otorhinolaryngol Ital..

[13] Zainine R, El Aoud C, Bachraoui R (2011). The plunging goiter? about 43 cases. Tunis Med..

[14] Mosaddaque Iqbal S, Memon IM (2012). Thyroidectomy in Substernal Goitre: our view point. Pak J Surg..

[15] Rodrigues J, Furtado R, Ramani A (2013). A rare instance of retrosternal goitre presenting with obstructive sleep apnoea in a middle-aged person. Int J Surg Case Rep..

[16] Ntyonga-Pono M, Nzouba L, Mathias A (1998). Le goitre endothoracique toxique: à propos d'un cas. Médecine d'Afrique Noire.

[17] Rodriguez JM, Hernandez Q, Piñero A (1999). Substernal goiter: clinical experience of 72 cases. Ann Otol Rhinol Laryngol..

[18] Grainger J, Saravanappa N, D'Souza A (2005). The surgical approach to retrosternal goiters: the role of computerized tomography. Otolaryngol Head Neck Surg..

[19] Shai SE, Chen CY, Hsu CP (2000). Surgical management of substernal goiter. J Formos Med Assoc..

[20] White ML, Doherty GM, Gauger PG (2008). Evidence-based surgical management of substernal goiter. World J Surg..

